# Surgery

**Published:** 1859-07

**Authors:** 


### Bi-Monthly Periscope.

### SURGERY.

1. Popliteal Aneurism Treated by Compression and Manipulation. Under the care of *Mr. Thomas P. Teale.*—Joseph M., aged forty-eight, a laborer, who had been discharged from the army five years ago, was admitted into the Leeds Infirmary, on October 26, 1858. Three months before admission he first observed a slight swelling of his left leg, which was soon followed by pain around the knee and along the calf. He attributed his ailment to a sprain received the day before while he was exhibiting to his companions a piece of military drill, consisting of arching the trunk forward and backward so as to touch the ground with his hands in both directions. Two or three days afterwards he discovered a small prominence, like a marble, in the ham, which has continued to increase until the present time. It is a remarkable fact that during the Crimean war Mr. Teale had at one time in the hospital three cases of popliteal aneurism, the subjects of them all being militia men. On the admission of M. into the Infirmary, an oval tumor, about the size of a small lemon, occupied the left ham, and exhibited the usual signs of aneurism.

October 26.—On this day at noon, treatment by pressure of the artery as it passes over the pubes was commenced by means of the fingers, several students having kindly undertaken the treatment in turns.

30th.—This treatment was continued during twelve hours of the day, and left off during the night, until the present date. The tumor has become a little smaller, and perhaps harder; but as the improvement is not greater than has been frequently derived from mechanical pressure, the gentlemen were released from their compressing duties, and pressure by the ring tourniquet at the upper and middle portions of the thigh was substituted for manual pressure. The patient was taught the course of the artery, and was directed to vary the seat of pressure from time to time as the part became painful. He was, morever, instructed to leave off the pressure at night, so as to secure sufficient rest.

November 2.—Size of tumor not much lessened, but its walls feel thicker; pulsation is somewhat less, and is controlled by a slighter amount of pressure. At the outer side of the knee a branch of an artery can be felt pulsating.

5th.—Another enlarged anastomosing branch felt at the inner side of the knee.

9th.—Tumor slightly diminished in size, and more firm to the touch.

12th.—Not much change since the last report. Mr. Teale now made an attempt to disturb the fibrin within the sac by moderate manipulation.

14th.—No material change since the last report. Mr. Teale repeated the manipulation with much greater freedom, kneading the tumor in various directions. This was done at 1 P.M.

15th.—An hour and a half after the last manipulation, the tumor had entirely ceased to pulsate, and had become a solid mass.

26th.—The tumor having rapidly decreased in size, the patient left the hospital this day cured, having been able to walk about the ward for a few days.

Mr. Teale, addressing the students, made the following observations on this case: "You would observe, gentlemen, that on the seventeeth day of treatment, at which time the aneurism had been very slowly diminishing in size, and increasing in solidity, but still retaining a rather strong pulsation, I resorted to a new method of treatment, namely, manipulation of the tumor, in the hope of hastening the cure. For the introduction of this new element in the treatment of aneurism, we are indebted to Mr. Fergusson, who, in 1856, brought the subject under the consideration of the Medico-Chirurgical Society. This gentleman referred to cases in which 'spontaneous cure' was brought about apparently by the displacement of fibrin within the sac. He proposed to imitate nature in this respect, and by manipulation of the aneurism to displace portions of fibrin, which might obstruct the distal end of the aneurism, and might also, by the irregularity of surface, resulting from displaced masses of fibrin, afford new points for more speedy deposition, and the consequent consolidation of the aneurism. The treatment was adopted by Mr. Fergusson in two cases of right subclavian aneurism. Following his example, on November twelfth, I manipulated the aneurism by a kneading kind of pressure with the fingers. No particular result followed. On November fourteenth, at 1 P.M., I repeated the manipulation much more forcibly, and at 2·30 the same day, the tumor had become perfectly consolidated, and had ceased to pulsate. No further treatment was adopted. Absorption went on rapidly, and the patient left the hospital cured on November twenty-sixth, or thirty-one days after the beginning of treatment, and twelve days after the second manipulation. I think it is impossible, in this case, not to connect the sudden solidification of the tumor with the free manipulation practiced an hour and a half before. So strongly do I believe that the ultimate cure was owing to this proceeding, that I intend hereafter to put this mode of treatment, in cases of aneurism of the limbs, to the test of further trial. But, gentlemen, let me guard you against adopting this practice in aneurism of the carotids or of the innominate artery; or, in other words, in aneurism of arteries leading to the brain. In such cases you might run the risk of detaching small portions of fibrin, which, being carried along the current of blood to the brain, might then produce the serious mischief of that organ, and the consequent paralytic affections described by Dr. Kirkes in a paper of great value published in the *Medico-Chirurgical Transactions* for 1854. And this is not a mere speculative evil, as you will perceive from the following case which I will briefly notice, and which I regret has not been published in an extended form. In the year 1847, I was one of a numerous consultation on a doubtful case of carotid anuerism. The subject of it was a middle-aged female, in good health in other respects. She was seated in a chair while the tumor was examined by several persons in succession, and subjected by them to repeated handling and compression. While this was going on, she suddenly became pale and slipped off the chair. On being raised, she was found to be hemiplegic. After lingering in this state a few weeks she died. The tumor was found, after death, to be aneurismal. It was not, however, until I read the paper by Dr. Kirkes, that I fully appreciated the pathological bearings of this case. Now that we do understand it, let me urge upon you to bear it in your minds, and let it be a warning to you, in carrying out Mr. Fergusson's valuable suggestion, to limit the treatment by manipulation of the tumor to aneurism of the extremities. I may also add that, in one of the two cases of aneurism of the subclavian artery which are published by Mr. Fergusson, hemiplegia occurred very shortly after manipulation. It is not improbable that the aneurism in this case extended to the innominate artery. Mr. Fergusson alludes to the possibility of the hemiplegia being produced in the manner explained by Dr. Kirkes."—(*Medical Times and Gazette*, March 12, 1859.)

2. ON LABIAL CANCER. By *Professor Riberi.* (Omodei's Annali, vol. clxvi. p. 331.)—In a notice of the forthcoming third volume of Professor Riberi's "Lezioni Orali," an account is given of his experience with respect to labial cancer at the Turin Clinic. The *ages* of the 81 patients were as follows:—2 between twenty and thirty, 3 between thirty and forty, 11 between forty and fifty, 28 between fifty and sixty, 20 between sixty and seventy, and 17 between seventy and eighty. Of these, 69 belonged to the *peasantry* class, a predominance perhaps attributable to their unnutritious food, their abuse of peppers, garlic, vinegar, and the like condiments, their neglect of personal cleanliness, and their exposure to vicissitudes of the weather. Another predilection of the disease was for the *male* sex and the *lower* lip, inasmuch as only three of the cases occurred in women, and in only four instances was the upper lip affected, two of these occurring in men and two in women. In all but one patient the sanguineous temperament was manifested in a greater or less degree, showing the influence of the conditions of the blood-vessels and of the blood in this disease as compared with that of the nervous system. In seventy-six of the subjects the constitution was good, robust, or even athletic. This confirms the observation made by Pravaz, that the general belief is erroneous which supposes that lymphatic, delicate, cachectic constitutions, are most liable to cancer. Persons become cachectic and enfeebled as the disease advances, as its result, not as its cause.

In most of the patients an unhealthy state of the skin prevailed, and there were few cases in which some complication was not observed, arising from disturbances of the respiratory or circulatory organs, varix, varicose ulcers, chronic gastro-hepatitis, pellagra, etc. After awhile, the *glands* in the vicinity enlarge, and it is of importance to determine whether their increase be merely sympathetic or symptomatic of invasion of the disease. In the former case, a single gland only usually becomes enlarged, being of recent origin, round or oval in form, movable, and liable to spontaneous changes in size; it is painful and tender to the touch, the skin being warmer than usual, and in some cases slightly reddened. In symptomatic enlargement, two or more glands are almost always affected, large and indurated lymphatic cords stretching between them, and often down the side of the neck. After awhile, the glands may acquire a large size, assuming an irregular form, becoming more or less fixed at their base, being but slightly movable, and not undergoing spontaneous change in size.

Before proceeding to the operation, M. Riberi submits his patients to hygienic and medical treatment calculated to relieve any complication or subdue any inflammatory action that may be present. Some cases of cancroid would indeed be cured by such procedures had the patients sufficient patience to await the result. Believing the employment of caustics mischievous in almost all other forms of cancer, M. Riberi regards them as of great utility in epithelial cancer, especially of the face, when the base is small enough to admit of its entire destruction. But as the tissue of the lip is very soft and yielding, and cancer soon sends widely-spread roots into it, and as patients usually do not apply until the lesion has thus become extensive, the employment of caustics is not admissible. Moreover, considerable deformity may result from its application, and an aggravation of the disease may be produced when the whole has not been extirpated. The operation with the V incision, having its base toward the labial edge, and conjoined when necessary with cheiloplasty, is that to which Professor Riberi gives the decided preference. He enters into considerable details upon this part of the subject, for which we have not space. Whatever form of the operation be adopted, he insists upon the necessity of removing during its performance all glands that may be symptomatically affected.

Of seventy-eight persons operated upon, seventy-three left the clinic cured; some of these, however, returned at the end of more or less long periods suffering from other cancerous diseases, two succumbed to a reproduction of the disease while in the clinic, and three died after the operation from causes not connected with it. The following are the conclusions drawn from a consideration of the cases of the eighty-one patients:—1. The disease almost always commences as epithelial cancer or epithelioma of the skin or mucous surface of the lip, spreading thence to the parenchyma, and very rarely begins in this last, extending thence to the surfaces. 2. The skin is almost always primarily affected, and only in some rare instances by morbid diffusion from the mucous surface. 3. Although very frequently unaffected at first, the mucous membrane becomes almost always implicated in the course of the disease. 4. The cellular tissue of the parenchyma is always simultaneously affected, as are very frequently the mucous and sebaceous crypts, to the great number of which in the lips Benjamin Bell attributed the frequency of labial cancer. 5. The muscular tissue is sometimes unaffected, sometimes participates slightly in the disease, and in some cases is so involved as to become entirely destroyed. 6. Whatever our nosological distinctions may be in respect to the species of cancer, nature shows how ill founded they are, by exhibiting more than one of these together; but facial cancers are those in which this junction is seldomest observed.—(*British and Foreign Medico-Chirurgical Review*, April, 1859.)

